# Supplementary material for: Falling through the cracks: Increased vulnerability and limited social assistance for TB patients and their households during COVID-19 in Cape Town, South Africa
Source: PLOS Glob Public Health. 2022 Jul 28;2(7):e0000708. doi: 10.1371/journal.pgph.0000708 (PMC10021457; doi:10.1371/journal.pgph.0000708)
Supplement: Attachment — Submitted filename: Response to reviewers_Falling through the cracks_070622.docx [file pgph.0000708.s001.docx]

Dear Editor and Reviewers

Thank you for the opportunity to revise our paper PGPH-D-22-00517
“Falling through the cracks: increased vulnerability and limited social assistance for TB patients and their households during COVID-19 in Cape Town, South Africa.”

We thank both the editor and the reviewers for their fast response and constructive comments that have allowed us to improve the paper for publication.

Please find our point by point response below. We also include a copy of the revised manuscript with tracked changes to illustrate the changes made, as well as a clean copy.

We hope that these changes have much improved the paper to meet the publishing requirements of PLOS Global Public Health.

Sincerely,

Lieve and the research team

**JOURNAL REQUIREMENTS**

| **COMMENT** | **RESPONSE** |
| --- | --- |
| 1. Your co-author, Wanga Zembe-Mkabile [-wanga.zembe@mrc.ac.za](mailto:-wanga.zembe@mrc.ac.za), has not confirmed authorship of the manuscript. We have resent them the authorship confirmation email; however please check that the above email address for them is correct and follow up personally to ensure they confirm. Please note that we cannot proceed your manuscript until we have received confirmations from all co-author. | Dear Editor, thank you for picking this up. Dr Zembe has now confirmed authorship. |
| 2. Please note that your Data Availability Statement is currently missing the repository name and direct link to access each database. If your manuscript is accepted for publication, you will be asked to provide these details on a very short timeline. We therefore suggest that you provide this information now, though we will not hold up the peer review process if you are unable. | Dear Editor, as mentioned in our data availability statement, we cannot publicly share the data as study participants did not give consent to share the anonymised data publicly after the study. The original application to the ethics committee did not include an explicit request to participants to share data. Anonymised data can therefore not be shared in a repository but can only be made available after reasonable request to the SAMRC Ethics Committee for study replication. The contact details for the SAMRC Ethics Office are the following:  Research Integrity Office Secretariat  Ms A Labuschagne E-mail: [Adri.Labuschagne@mrc.ac.za](mailto:Adri.Labuschagne@mrc.ac.za) |
| Please review your reference list to ensure that it is complete and correct. If you have cited papers that have been retracted, please include the rationale for doing so in the manuscript text, or remove these references and replace them with relevant current references. Any changes to the reference list should be mentioned in the rebuttal letter that accompanies your revised manuscript. If you need to cite a retracted article, indicate the article’s retracted status in the References list and also include a citation and full reference for the retraction notice. | Dear editor, we have checked all references on the Retraction Watch Database and none of them returned as retracted. |
| [NOTE: If reviewer comments were submitted as an attachment file, they will be attached to this email and accessible via the submission site. Please log into your account, locate the manuscript record, and check for the action link "View Attachments". If this link does not appear, there are no attachment files.] | Attachments have been checked |
| While revising your submission, please upload your figure files to the Preflight Analysis and Conversion Engine (PACE) digital diagnostic tool, [https://pacev2.apexcovantage.com/](https://protect-za.mimecast.com/s/phDLCj2gGMIYxM4rsnHlZr). PACE helps ensure that figures meet PLOS requirements. To use PACE, you must first register as a user. Registration is free. Then, login and navigate to the UPLOAD tab, where you will find detailed instructions on how to use the tool. If you encounter any issues or have any questions when using PACE, please email PLOS at [figures@plos.org](mailto:figures@plos.org). Please note that Supporting Information files do not need this step. | Fig 1. Conceptual framework and Fig 2. Determinants of vulnerability have both been uploaded to PACE. |
| Please include the following items when submitting your revised manuscript:   - A rebuttal letter that responds to each point raised by the editor and reviewer(s). You should upload this letter as a separate file labeled 'Response to Reviewers'. - A marked-up copy of your manuscript that highlights changes made to the original version. You should upload this as a separate file labeled 'Revised Manuscript with Track Changes'. - An unmarked version of your revised paper without tracked changes. You should upload this as a separate file labeled 'Manuscript'. | We have uploaded the following documents:   - Response to reviewers - Revised manuscript w track changes - Manuscript |

**REVIEWER #1: ASSESSMENT**

| **COMMENT** | **RESPONSE** |
| --- | --- |
| □ Topical relevance and novelty of the work: This manuscript is relevant to the journal to which it is submitted. The topic is relevant to the general understanding of the effects of the current socioeconomic situation on the treatment of one of the most prevalent infectious diseases, TB and suggests a solution to rectify it. | Thank you |
| □ Background It sets the stage for understanding the origins and importance of the work. | Thank you |
| □ Aims The primary aim and secondary aims are clearly stated. | Thank you |
| □ Methods The cross-sectional phenomenological exploratory study method is appropriate to address the stated aims. | Thank you |
| □ Statistics As it a qualitative study, there is no need to apply the statistical methods. | Thank you |
| □ Results The results are presented in a logical way, and they address the stated aims. | Thank you |
| □ Discussion The discussion addresses the results in a systematic way, pointing out the strengths and weaknesses of the study. I would have liked to know, how many of these 15 patients were HIV positive and how many were on antiretroviral (ARV) therapy. This data is not available. | Dear reviewer, thank you for making this point. Unfortunately, we did not ask patients for their HIV status as our focus was on TB, and we can therefore not report on this. |
| □ Limitations The limitations of the study, its design, its methods and reported data are clearly indicated by the authors. There are no ethical concerns. | Thank you |
| □ Figures and Tables The included figures and tables are appropriate for the manuscript. The tables are easy to read and understand. The legends for the figures and tables are appropriate and easy to read. | Thank you |
| □ References There are appropriate number of timely references that support the background and discussion. | Thank you |
| □ Title/Keywords These are appropriate for the manuscript. | Thank you |
| □ Abstract The abstract captures the most salient points of the study including key results and conclusions. | Thank you |
| □ Readability The manuscript is easy to read, and the use of language is appropriate. Following are the specific minor areas where I offer corrections for grammar, spelling, or punctuation corrections: Page 23 Line 351 Use word “people” instead of short form used “ppl” Page 28 Line 475 Use upper case F for the first word in the sentence “for” | Dear reviewer, thank you for picking up these spelling mistakes. We have corrected them. |
| □ Approval I have noted that appropriate research approval was obtained. | Thank you |
| □ Conflicts There are no conflicts of interest, and it is clearly stated. | Thank you |
|  |  |

**REVIEWER #2: SUMMARY**

| **COMMENT** | **RESPONSE** |
| --- | --- |
| MAJORS ISSUES |  |
| Introduction There are sections with statements that lack referencing. | Dear reviewer, thank you for this comment. I have added more references: line 78, 86, 87, 92, 100, Figure 1, line 122, 125, 129, 136, 138, table1, |
| Line 52 and 53 says the patients were turned away but it doesn’t indicate who turned them away. | Dear reviewer, thank you for picking this up. This statement was actually in the wrong place as it refers to the situation in South Africa (SA), not globally. I have therefore deleted the sentence in line 52 and 53. The gist of this statement is included in line 68-70 which relates to the situation in South Africa and includes the reference. |
| What do other studies say about loss of income and support systems, and health impact to TB patients and their households in the context of Covid 19 if any? | Dear reviewer, we have added more content on other studies looking at the health impact, loss of income and increase in costs of COVID-19 for TB patients. However, we could not find any studies investigating the impact of COVID-19 on social support of TB patients.  We have added the following sections:  “The biomedical interactions and co-infections between TB and COVID-19 (24-27), as well as the impact of the COVID-19 pandemic on the TB epidemic in terms of incidence, TB diagnosis and enrolment on treatment, and disruption of health services for TB patients has been widely described, discussed and modelled (7, 28-33).”  “This corresponds with findings from the few available studies on the financial impact of COVID-19 on TB patients. A study in India found that the number of TB patients’ households with zero household income increased from 6% before lockdown to 54% during the strict lockdown (38). The study further found indications that the income loss for households and poor restoration of income after lockdown will have had a serious impact on the nutrition of the TB patients and families. A study in Cairo, similarly, found that the early COVID-19 lockdown was associated with a rise in catastrophic costs for TB patients (39).”  “The loss of social support has been reported as one of the major consequences of COVID-19, and particularly its effect on mental health and isolation (49-52). The effect of COVID-19 on social support for TB patients and the resulting loss of material support, however, has to our knowledge not been studied.” |
| Methodology While the sample size is logical, it is not clear as to how the researchers got to the sample of 15 participants. Did they reach saturation by the 15th patient or they just took a logical number? | Thank you for this comment. We chose a sample size of 15 interviews prior to commencement of the study based on several factors as described in the revised paragraph below:  “A sample size of 15 interviews was chosen for the study. We followed Malterud’s model to ascertain information power to guide the sample size (23). According to the model, considerations about study aim, sample specificity, theoretical background, quality of dialogue, and strategy for analysis should determine whether sufficient information power will be obtained with less or more participants included in the sample (23). As the aim of our study is narrow, the combination of participants is highly specific for the study aim, the study is supported by established theory, the interview dialogue is strong, and the analysis includes in-depth exploration of narratives, a smaller sample size is warranted. In addition, findings from a recent study showed that a sample size of 9-17 interviews is sufficient to reach saturation for relatively homogenous study populations (24), and previous research by the authors with the same study population reached saturation around 15 interviews (17).” |
| Most TB diagnosis, treatment and treatment review in the public health centres are managed by the nurses and not all TB patients get referred to the doctors. What was the inclusion and exclusion criteria for the participants? | I have added the inclusion/ exclusion criteria:  “We requested nurses to refer any patient older than 18 years and on treatment for at least two weeks to the research assistant in the adjoining room after their check-up.” |
| In the data collection, the researchers noticed and considered new areas for further interviews. Did they follow this up with the participants? | New areas were noticed and noted for further research. We have corrected the section as follows:  “Following each day of interviews, LV and WZ discussed the interviews, noting initial thoughts and meanings, refining questions, and noting emerging areas for further research.” |
| It is not clear how the researchers ensured accuracy of the translation without any Bias as there was only one bilingual researcher for the research context who had the sole responsibility of translation. | The transcription and translation was performed by a professional translation company, and checked for accuracy by the bilingual researcher. We have revised the sentence as follows:  “All interviews were recorded, transcribed verbatim and translated into English by a professional translation company and checked against the original recording to ensure accuracy by the bilingual researcher.” |
| Results Line 242,243 &244. The researchers indicate that about half of the participants were admitted to hospital for long Periods for severe illness and lost out on income as a result. This is conflicted by table 3 as it shows that only one patient lost income due to TB illness. | Thank you for picking up these inconsistencies. We have looked through the data again and realized that the confusion comes in with the difference between employment and income. Participants were asked about employment in the participant profile form which was administered before the interview. Some participants, however, indicated in the profile form that they are unemployed but would report during the interview that they have a small income from informal work. Hence, while they didn’t strictly lose their employment as they were unemployed, they lost out on income from informal work. We have therefore deleted the rows in the table referring to employment and replaced them with information regarding income as this is the central argument of the section:  - TB patients that lost income due to TB, COVID-19 or both (formally or informally)  - household members of TB patients that lost income due to TB, COVID-19 or both (formally or informally)  In addition, we have revised the concerning paragraphs as follows:  “Most participants were unemployed but some earned a little cash from informal work such as handyman, collecting recycling, guarding parked cars, braiding hair, and selling chicken feet and ginger beer. This income, however, was lost for those participants that became too ill to work. In addition, those that hadn’t already lost their income due to TB, now also lost their income due to the COVID-19 restrictions. One participant had lost his formal employment while five other patients reported to have lost income either due to the TB illness, COVID-19 restrictions or both. For example, one of the participants earned a little income braiding hair but found she was too tired to braid hair when she was sick with TB. When she started getting better, she struggled to get customers due to COVID-19. “  *“They (customers) are not coming as before because people don't have money - a lot of people - due to this COVID, so people do their hair very seldom.” (female, 32 years old)*  “In addition to their own loss of income, six participants mentioned one or more members of their household losing income, severely impacting the household budget. With many participants relying on their household during their illness, the loss of income in the household affected them as well.” |
| Line 247 indicates that several participants working informally lost income due to lockdown restrictions while table 3 shows only 2 participants. |  |
| MINOR ISSUES The article needs editing. | We have given the manuscript a thorough editing and have made several changes to improve grammar and readability |
| Acknowledge the sources of the data e.g. table 1 For the headings in table 1. Please put a dash if there is no information to fill in or a zero if appropriate. | We have added the source of the data for table 1. I also adjusted the amounts and number of beneficiaries in that table as I realized that I had previously put in the amounts and number of beneficiaries for the next financial year (April 2021). I have now replaced them with data for the correct period i.e. February - March 2021 during which the interviews were conducted.  In addition, we have revised all tables and added a dash or zero where appropriate. |
| Consent forms. It’s the participants who are supposed to give informed consent after explanation by signing the consent form. | We have revised the sections related to the signing of consent as follows:  Line 163-164. “If the patient agreed to participate in the study, the patient was asked to sign the consent form and a pre-interview participant profile form was completed with the patient.”  Line 191-199. “All participants were given an information sheet and informed consent form which was read together with the participant and explained in detail. Participants were informed about the purpose of the study, procedures involved, risks and benefits of the study, and their rights as participants. The right to decline participation was emphasised, as well as an assurance given that the decision not to participate would not affect the services they receive at the clinic or any other government institution. Participants were given an assurance of confidentiality and strict protection of collected data. Following the detailed explanation, participants were asked to sign the consent form. All 15 participants in the study signed the consent form.” |
| Line 109 Covid 19 pandemic not “epidemic.” | The word epidemic was replaced with pandemic. |
